# Brachium Pontis Gliosarcoma With Well-Differentiated Cartilaginous Tissue: A Case Report

**DOI:** 10.1097/MD.0000000000001735

**Published:** 2015-10-23

**Authors:** Lei Wang, Yuanyang Xie, Yan Liu, Jun Tan, Zhongliang Chen, Yu Xiao, Ying Xia, Zefeng Peng

**Affiliations:** From the Department of Neurosurgery, Xiangya Hospital Central South University, Changsha (LW, YX, JT, ZC, YX, ZP); Department of Neurosurgery, Affiliated Haikou Hospital Xiangya School of Central South University, Haikou (LW, YX); and Department of Neurology, Changsha City Central Hospital, Changsha, China (YL).

## Abstract

Gliosarcoma (GS) belongs to World Health Organization grade IV neoplasm and displaying glial and mesenchymal differentiation. Only rare cases of GS have been reported in the brachium pontis and 4th ventricle region. Here, we report a rare case of GS located on brachium pontis region and extending into the 4th ventricle with well-differentiated cartilaginous metaplasia. A 28-year-old male patient experienced intermittent headache, vomiting, and gait disorders for 3 months. Magnetic resonance imaging (MRI) showed a heterogeneous ring-enhancement lesion with weak central enhancement in left brachium pontis and 4th ventricle region. Histology revealed the GS was constituted with glial and sarcomatous elements. After immunohistochemical analysis, a diagnosis of GS with cartilaginous differentiation was then made.

Symptoms of GS, including headache, aphasia, hemiparesis, cognitive decline, and seizures, mainly determined by the location. The clinical manifestation and radiologic characteristic is not significantly different from that of glioblastoma. The grade of resection is the significant factor related to prognosis of GS, and the clinical effect of adjuvant radiotherapy and chemotherapy need further study. Reporting additional cases would be of great help in better understanding of this location and pathologic type of GS.

## INTRODUCTION

Gliosarcoma (GS) is a rare form of primary central nervous system (CNS) malignant neoplasm consisting of malignant glial cells mixed with sarcomatous components. GS belongs to World Health Organization grade IV neoplasm and displaying glial and mesenchymal differentiation according to the 2007 World Health Organization classification of tumors of the CNS.^1^ GS is relatively infrequent clinically and accounted for 2% to 8% of all glioblastomas (GBMs),^1–3^ occurs commonly in the cerebral hemispheres, mostly located on temporal lobe, parietal lobe, frontal lobe, and occipital lobe.^2,4–7^ This kind of tumor is extremely rare at brachium pontis and 4th ventricle. Herein, we report a case of primary GS with a cartilaginous component located on left brachium pontis region and extending into the 4th ventricle in a 28-year-old male presenting with headache, vomiting, and walking unstable.

## CASE REPORT

**FIGURE 1:**
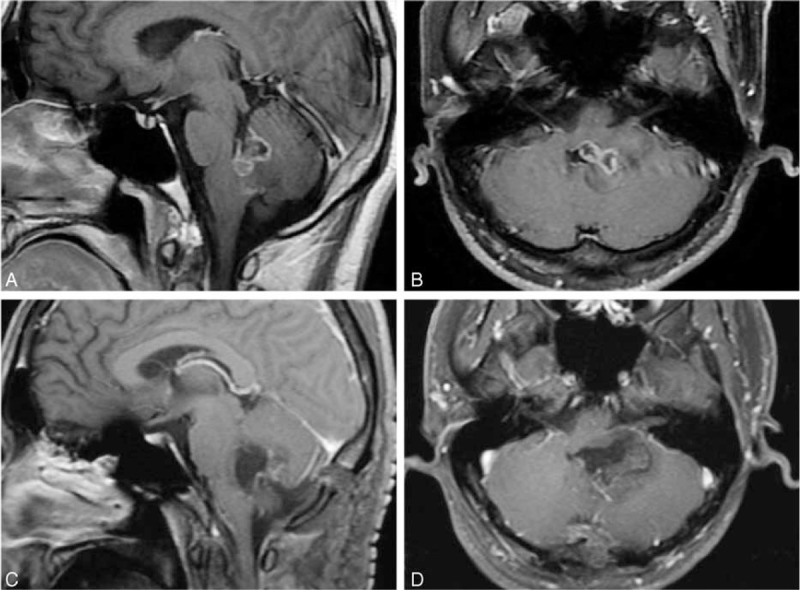
Preoperative sagittal (A) and axial (B) Gd-enhanced T1-weighted MR images demonstrating a heterogeneous ring-enhancement lesion involving left brachium pontis region and extending into the 4th ventricle. Sagittal (C) and axial (D) Gd-enhanced T1-weighted MR images obtained on the second day after surgery evaluation with no evidence of tumor. Gd = gadolinium, MR = magnetic resonance.

A 28-year-old male adult experienced with intermittent headache, vomiting, and walking unstable for 3 months. Magnetic resonance imaging (MRI) showed a 2.2 cm × 3.6 cm × 3.5 cm heterogeneous ring-enhancement lesion with weak or unremarkable central enhancement in left brachium pontis and 4th ventricle region. MR images showed hypointense on T1-weighted MR images and enhancement on T1 gadolinium (Gd) image (Fig. 1A, B).

The patient underwent a surgical exploration via retrosigmoid sinus approach. During surgery, a discrete, texture medium, grittiness mass with associated chondroosseous portions in the left brachium pontis and 4th ventricle was encountered. There was no adherence or attachment to the pia or dura. The tumor was microscopically totally removed confirmed by Gd-enhanced T1-weighted MRI views 2 day postsurgery (Fig. 1C, D) and subjected to histopathological examination. After formalin fixation, the tumor samples routinely with HE and reticulin. Immunohistochemical (IHC) staining was performed with monoclonal antibodies against glial fibrillary acidic protein (GFAP) (1:500), S-100 (1:300), CD68 (1:500), and epithelial membrane Aantigen (EMA) (1:250), respectively.

**FIGURE 2:**
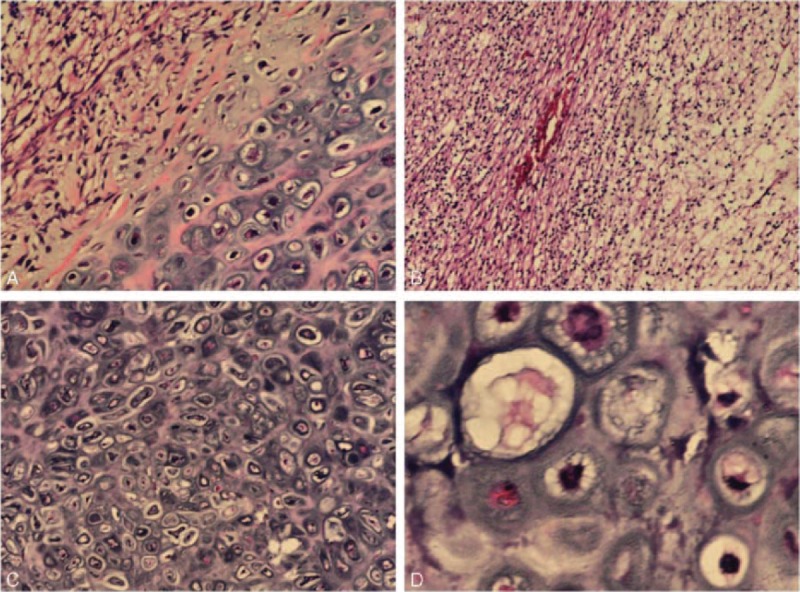
(A) Photomicrographs revealing cellular components of the GS (H&E, ×100). (B) Gliomatous area of the tumor with features of a GBM (H&E, ×400). (C) Sarcomatous area of the tumor with features of a cartilage (H&E, ×100). (D) Areas of cartilage tissue (H&E, ×400). GBM = glioblastoma, GS = gliosarcoma.

**FIGURE 3:**
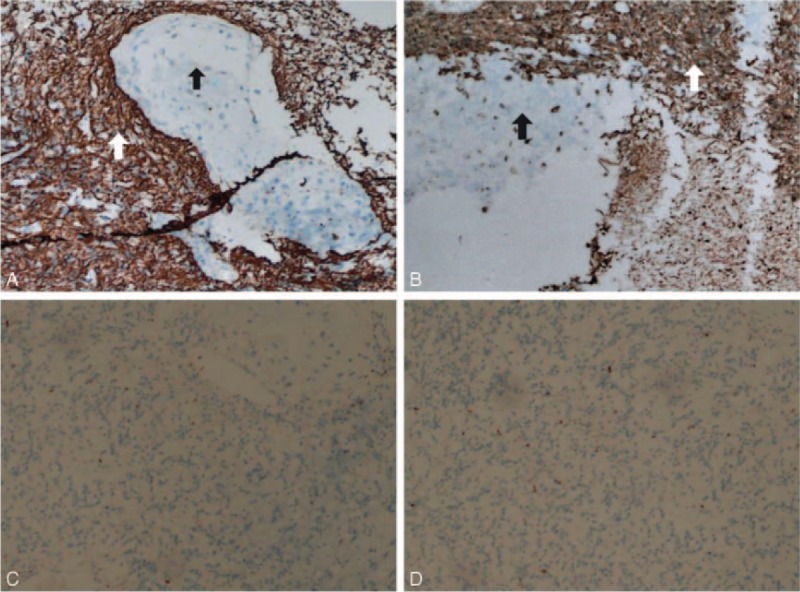
(A) Immunohistochemistry for GFAP highlighting the neoplastic glial component, GFAP negative sarcomatous area with cartilage tissue (GFAP, ×100). (B) Immunohistochemistry for S-100 highlighting the neoplastic glial component, S-100 negative sarcomatous area with cartilage tissue (S-100, ×100). (C, D) Both components were negative for CD68 and EMA (CD68, EMA, ×100). EMA, epithelial membrane Aantigen; GFAP, glial fibrillary acidic portein

Microscopically, there was tumor with 2 morphologically distinct elements in formalin-processed paraffin-embedded tissue (Fig. 2A). The first was characterized by typical astrocytic tumor cells composed of proliferating astrocytes and gametocytes with cytoplasmic vacuolation (Fig. 2B). Another kind appeared clusters of numerous oval and round shape cells with malformed and densely nucleus. Well-differentiated cartilage island was observed. The tumor cell nests are separated from each other by abundant myxoid stroma (Fig. 2C). By immunohistochemistry, the glial component showed strong and diffuse cytoplasmic GFAP and S-100 protein positivity (Fig. 3A, B, white arrow). On the contrary, the oval and round cell part of the tumor was GFAP, S-100 protein negative (Fig. 3A, B, black arrow). Both components were negative for CD68 and EMA (Fig. 3C, D).

## DISCUSSION

GS, which comprise 2% to 3% of GBMs, are rare tumors of the CNS. They are most commonly occurred in the frontal, temporal, and parietal lobes in patients older than 60 years of age. Symptoms of this tumor, including headache, aphasia, hemiparesis, cognitive decline, and seizures, mainly determined by the location. Also the clinical manifestation and radiologic characteristic is not significantly different from that of GBM. To the best of our knowledge, only rare cases of GS have been reported in the brachium pontis and 4th ventricle region. GS shows a predilection for a superficial location and dural invasion and thus may not be considered in the initial workup of parenchymal masses.^8^ There were 2 cases of primary GS of the posterior cranial fossa in patients older than 60 years of age have been reported. In these cases, authors suggest that the atypical radioimaging characteristics of GS do not allow diagnosis to be made because the limited cases and the variability of its imaging finding, so the definite diagnosis may be apparent only upon biopsy of the lesion.^9^ Consistent with previous reports, our case also illustrates that GS could be occurred in deep-seated brain lesions, such as brainstem and 4th ventricle that present without dural involvement. However, the patient in our case was younger than 30 years.

GS was constituted by glial and sarcomatous element, the glial component belongs to GBM and the sarcomatous element having the features of any sarcoma type. The sarcomatous components derived from the neoplastic transformation of the hyperplastic tumor blood vessels from the past main research literature.^10^ Recent reports suggest a monoclonal origin of both components of GS, with the sarcomatous component originated from aberrant mesenchymal differentiation of the glioma.^11^ The common sarcomatous components also include malignant fibrous histiocytoma,^2,12,13^ fibrosarcoma,^5,14^ rhabdomyosarcoma,^15^ angiosarcoma,^16^ osteosarcoma,^17^ etc.^18^ The diagnosis of GS rests on the exclusion of GBM associated with reactive leptomeningeal or vascular changes and sarcomas with entrapped reactive astrocytes, diagnosis requires identification of a malig glial (usually astrocytic) component and at least one X100 magnification field of a sarcoma histologically consistent with a malignant fibrous histiocytoma or fibrosarcoma.^19^

Histopathology showed features of proliferating astrocytes with marked nuclear atypia, frequent mitotic figures, pseudopalisading necrosis, tumor giant cells, and angiogenesis. The neoplastic glial cells were intensely positive for GFAP and S-100 and the sarcomatous component negative for GFAP and S-100. CD68 and EMA showed negativity in both glial and sarcomatous elements. histomorphological features along with immunohistochemistry findings suggested a diagnosis of GS. Some literatures suggested osseous or cartilaginous component in GS may be indicate high malignant degree.^20–22^ Neoplastic astrocytes could also differentiate into cartilage through mucopolysaccharide deposition, which can evolve to form a chondroid matrix.^23,24^

The patient tolerated surgery and was discharged 2 weeks later. Because of postoperative hyperpyrexia and economic reasons, chemotherapy and radiotherapy were not given immediately at the time of hospitalization. Two months after discharge, there was no relapse of tumor shown by MRI evaluation. Additionally, the patient began to receive 60 Gy of radiation therapy and long-term follow-up is necessary. In terms of therapies and prognosis of GS, several literatures suggest that the prognosis of GS is similar than that of GBM.^25,26^ What is noteworthy is that tumor excision and adjuvant radiotherapy seem to better the prognosis.^26^ This survival difference depends largely on extent of resection performed, adjuvant radiotherapy, and chemotherapy administered to a limited number of patients did not improve survival in this study.^27^

In general, to the best of our knowledge the literature contains only rare case reports of GS located on brachium pontis region and extending into the 4th ventricle with well-differentiated cartilaginous metaplasia. Reporting additional cases would be of great help in better understanding of this location and pathologic type of GS.
